# Self-efficacy measurement instruments for individuals with coronary artery disease: A systematic review

**DOI:** 10.1371/journal.pone.0299041

**Published:** 2024-03-04

**Authors:** José Alexandre Barbosa de Almeida, Rêncio Bento Florêncio, Jéssica Costa Leite, Karolinne Souza Monteiro, Lucien Peroni Gualdi

**Affiliations:** 1 Rehabilitation Sciences Graduate Program, Federal University of Rio Grande do Norte, Santa Cruz, RN, Brazil; 2 Professor of department of Physical Therapy Graduate, Centro Universitário Natalense, Natal, RN, Brazil; 3 Professor of department of Physical Therapy Graduate, Centro Universitário UNIFACISA, Campina Grande, PB, Brazil; 4 Professor of department of Physical Therapy and Rehabilitation Sciences Graduate Program, Federal University of Rio Grande do Norte, Santa Cruz, RN, Brazil; Universidade Federal de Sao Paulo, BRAZIL

## Abstract

**Introduction:**

Over the past decade, there has been a heightened interest in evaluating self-efficacy among patients with coronary artery disease (CAD). A significant number of instruments have been developed and validated, yet the need remains to assess the quality of their studies and their properties.

**Objectives:**

To evaluate the measurement properties and link the content extracted from self-efficacy instrument items for individuals with CAD to the International Classification of Functioning, Disability, and Health (ICF).

**Methodology:**

The study was conducted following the Cochrane systematic review guidelines and COnsensus norms for Selection of health Measuring INstruments (COSMIN), registered under CRD42021262613. The search was carried out on MEDLINE (Ovid), Web of Science, EMBASE, and PsycINFO, including studies involving the development and validation of self-efficacy instruments for individuals with CAD, without language or date restrictions. Data extraction was performed in May 2022 and updated in January 2023 and all the steps of this review were carried out by two different collaborators and reviewed by a third when there were divergences. Modified Grading of Recommendations, Assessment, Development and Evaluation (GRADE) recommended by COSMIN was used to determine the quality of evidence as high, moderate, low, or very low. Instrument categorization was carried out per COSMIN recommendations, according to the construct of interest and study population into three categories (A, B, or C).

**Results:**

A total of 21 studies from 12 instruments were identified. The best-rated instruments received a recommendation of B, which means, additional validation studies are needed. Barnason Efficacy Expectation Scale (BEES) showed high-quality evidence for structural, construct, criterion, and internal consistency validity; Cardiac Self-Efficacy Scale (CSES) demonstrated high quality for content, structural, cross-cultural validity, and internal consistency; Self-efficacy for Appropriate Medication Use (SEAMS) achieved a high level for structural, criterion, and internal consistency validity; Cardiovascular Management Self-Efficacy Scale exhibited high-level validity for structural, criterion, construct, and internal consistency. The CSES showed content linkage with all domains of the ICF, as well as the highest number of linkages with the categories.

**Conclusions:**

Instruments with a B-level recommendation hold potential for use. More studies assessing measurement properties are needed to reinforce or improve these recommendations. The CSES stands out as the most comprehensive instrument concerning the ICF.

## Introduction

Self-efficacy is related to the individuals’ confidence in their ability to gather cognitive, motivational, emotional, and behavioral resources necessary to achieve a goal, deal with a specific situation, or perform a task [1]. It also encompasses elements of motivation, planning, organization, and awareness of skills necessary for dealing with illnesses, reflecting a sense of self-responsibility throughout pathological processes [1, 2]. Consequently, it becomes a crucial factor for health promotion and management of chronic conditions such as coronary artery disease (CAD) [1–3]. Moreover, it is linked to improved quality of life, mental well-being, and enhanced adherence to rehabilitation processes [4, 5].

Several general self-efficacy measurement instruments, whether or not related to specific disease conditions [6], assess specific individual conditions or behaviors associated with or without diseases (e.g., eating behavior, physical activity, and medication adherence) [7–9], or evaluate individuals in relation to their diseases (e.g., asthma, stroke, and coronary artery disease (CAD) [10–13]), are available in the literature. While these instruments are well-established, there is a need to assess their methodological rigor to guide the selection of the most suitable instrument for clinical practice and cardiovascular rehabilitation, considering their quality.

CAD remains one of the leading causes of mortality and morbidity worldwide [14]. It is estimated that by 2030, the current prevalence rate of 1,655 per 100,000 population will exceed 1,845 [14]. It is characterized by the onset of symptoms such as chest pain, dyspnea, and a sensation of pressure or tightness at varying levels of exertion. Conventional treatment involves the individual’s adherence to cardiovascular rehabilitation programs and lifestyle changes, aimed at delaying and preventing future complications, as well as improving physical fitness through resistance and aerobic training [15].

Since the level of self-efficacy can influence adherence to rehabilitation [16], it becomes necessary to have instruments that assess self-efficacy within this population, aiming to enhance treatment and rehabilitation programs adherence [1–3]. In this context, evaluating self-efficacy instruments for individuals with CAD may contribute to understanding the available tools for clinical practice and research, as well as assisting healthcare professionals in individual interventions.

Therefore, this systematic review aims to evaluate the clinimetric properties of self-efficacy instrument items for people with CAD and to relate their content to the International Classification of Functioning, Disability and Health (ICF) [17].

## Materials and methods

The systematic review was conducted *f*ollowing the Cochrane systematic review guidelines [18] and the COnsensus norms for Selection of health Measuring INstruments (COSMIN) [19–21] guidelines. The protocol was registered in the International Prospective Registry of Systematic Reviews (PROSPERO) under the registration number CRD42021262613 [22].

### Search strategy

The search was conducted in May 2022 in MEDLINE (Ovid), Web of Science, EMBASE, and PsycINFO databases, considering: (1) the construct of interest (cardiac self-efficacy); (2) target population (individuals with CAD); (3) type of instrument (questionnaire or scale); and (4) measurement properties; the latter was assessed using validated search filters for measurement studies previously applied in previous reviews and recommended by COSMIN [23].

Additional searches for relevant studies were conducted manually by checking the reference lists of primary studies and review articles. Searches were repeated prior to the final analysis in January 2023 using a date filter so that we could find studies published after our first search. The search strategies are provided in S1 File.

### Study selection

Studies that developed and validated clinimetric properties of self-efficacy measurement instruments for individuals with CAD were included, with no restrictions on publication date or language.

Clinical trials or validation studies using measures reported by third parties, theses, dissertations, and those published as abstracts were excluded. Additionally, studies of instruments that had self-efficacy as part of their construct (e.g., self-management, self-care, self-control) were also excluded, limiting the scope to self-efficacy instruments for patients with CAD or with participant reports of the same diagnosis in the study.

The search results were imported into the reference management tool Mendeley (https://www.mendeley.com). Duplicates were removed prior to the selection process, and the reference list was exported to the systematic review platform Rayyan Qatar Computing Research Institute (https://rayyan.qcri.org) [24].

Two independent authors (JABA and KSM) selected the studies simultaneously based on titles and abstracts. After this step, the same authors conducted independent and simultaneous full-text readings and documented reasons for excluding ineligible studies. In cases of disagreement, a meeting was held for discussion and consultation with a third reviewer (LPG).

### Data extraction

Data extraction was carried out by two authors (JABA and LPG) following COSMIN and Cochrane [18–20]. The extracted information included title, authors, year of publication, general instrument characteristics (construct, subscales, number of items, version, score), study design, target population, sample size, individual characteristics (e.g., age range, gender, research location, country, language, selection methods), and clinimetric properties. A third reviewer (KSM) was consulted for reviewing the extracted data.

### Study quality

The methodological quality of the studies was assessed by two independent authors (RBF and JCL) using the COSMIN RoB Checklist [25, 26]. This tool considers 10 measurement properties and consists of boxes with 3 to 35 items addressing aspects of comprehensiveness, relevance, and inclusiveness of the items included in the instrument. Each box assigns a methodological quality score for instrument development: (1) content validity, (2) structural validity, (3) internal consistency, (4) cross-cultural validity, (5) measurement invariance, (6) reliability, (7) measurement error, (8) criterion validity, (9) construct validity, and (10) responsiveness. Each item has four response options: inadequate (I), doubtful (D), adequate (A), and very good (V) [25]. Disagreements were resolved by a third author (KSM).

The content extracted from the measurement instruments was linked using the Comprehensive International Classification of Functioning, Disability and Health (ICF) Core Set following recommendations of Cieza et al. (2016) [17, 27–29] conducted by two separate authors (JABA and RBF). Subsequently, a third author (JCL) reviewed the contents in case of discrepancies.

### Data synthesis

Initially, a narrative synthesis of the results was prepared. In cases where the same instrument was validated for different populations, the assessment of measurement properties was performed considering a single instrument, with the particularity of each version being discussed. A combination of measurement properties determined the overall evidence of the instrument. Studies were grouped based on the similarity of instrument versions.

The results were assessed in groups or summarized in relation to the measurement property criteria to determine whether they were sufficient (+), insufficient (-), inconsistent (±), or indeterminate (?). The criteria were also subjectively evaluated by the reviewers (JABA and LPG), according to COSMIN criteria [19]. Additionally, the Modified Grading of Recommendations, Assessment, Development and Evaluation (GRADE) recommended by COSMIN was used to determine the quality of evidence as high, moderate, low, or very low [19, 30].

Subsequently, the instruments were categorized and justified according to COSMIN recommendations [21], considering the construct of interest and study population into three categories: (A) the instrument is recommended for use and the results are reliable; (B) when it can be recommended but requires further validation studies; and (C) the instrument should not be recommended due to insufficient properties.

## Results

**Fig 1:**
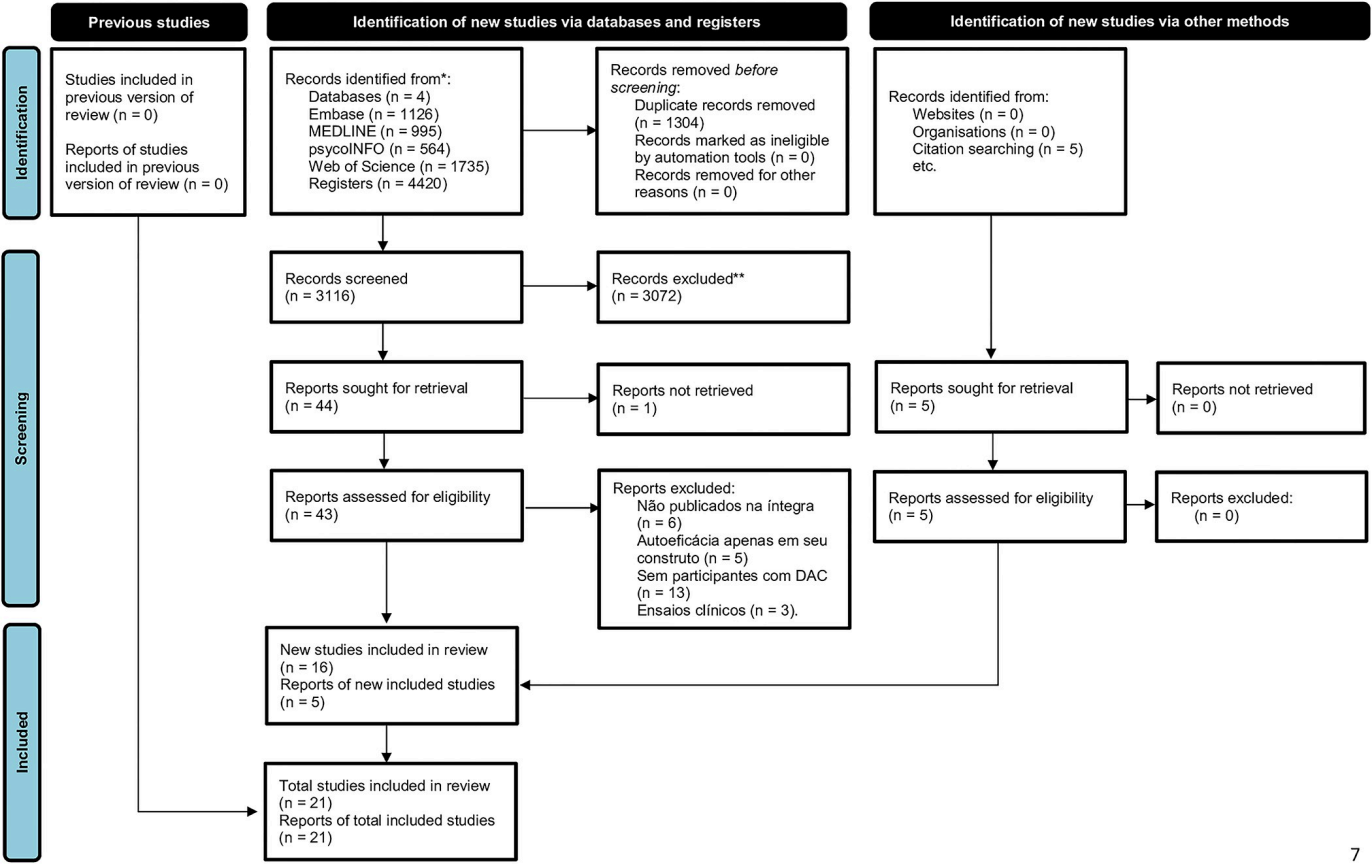
PRISMA flowchart of the study strategy.

A total of 4420 references were identified from the EMBASE (n = 1126), MEDLINE (n = 995), PsycoINFO (n = 564), and Web of Science (n = 1735) databases. 1304 duplicates were removed, leaving a total of 3116 for screening. Considering eligibility criteria, 3072 studies were excluded. Out of the remaining 44 studies, 28 were excluded due to not being available in full text (n = 6), having self-efficacy in only one of the instrument domains (n = 5), not including participants with a diagnosis of CAD (n = 13), and being clinical trials (n = 3), resulting in 16 studies included. Additionally, a search in the reference lists of included studies was conducted and 5 studies were included in the results, totaling 21 articles for the review (Fig 1).

### Characteristics of included studies and instruments

The included studies were published from 1998 to 2021. All of them aimed to develop or validate self-efficacy instruments specific to cardiovascular diseases or general ones that included individuals with CAD in their processes. The 21 included articles correspond to 12 self-efficacy measurement instruments in different versions available in the English language. Among the 21, 6 studies regards to instrument development, and 15 to validation.

The number of participants ranged from 18 to 726, totaling 5,393, with male predominance in 20 studies. The studies were conducted in the United States (n = 5) [12, 31–34], China (n = 3) [8, 35, 36], Australia (n = 2) [37, 38], Italy (n = 2) [39, 40], Jordan (n = 2) [41, 42], South Korea (n = 1) [43], Iran (n = 1) [44], Thailand (n = 1) [45], Sweden (n = 1) [13], and Brazil (n = 2) [46, 47]. The Brazilian studies refer to the same instrument, one aiming to adapt and assess measurement properties [47], and the other to evaluate construct validity [46]. Barnason et al. (2002) did not report the country where the study was conducted [48]. The number of items per instrument ranged from 8 to 22, and factor structures were seen as unidimensional, bidimensional, and tridimensional. Table 1 presents the general characteristics of the included studies.

**Table 1:** Characteristics of included studies.

AUTHOR (YEAR)	INSTRUMENT	STUDY DESIGN	POPULATION						INSTRUMENT				USED TOOLS
			N	AGE	GENDER	COUNTRY	LOCAL	LANGUAGE	APPLICATION METHOD	ITEMS	SCORE	SUB-DIMENSIONS	
**Barnason et al (2002)**	Barnason Efficacy Expectation Scale (BEES)	Development/ Validation	726	?	?	?	?	English	Interview	15	The scale is scored by summing the numerical ratings for each response to obtain a total score. The instrument’s total score ranges from 15 to 60	No	Jenkins’s Self-Efficacy Expectation; Medical Outcomes Study Short-Form 36 (MOS SF-36)
**Burke et al. (2003)**	Cholesterol-Lowering Diet Self-Efficacy Scale	Development/ Validation	272	Study 1: 59 ± 16.5; Study 2: 56 ± 11	Study 1: 33 M, 11 F; Study 2: 119 M, 109 F.	USA	Study 1: Cardiac Rehabilitation Program at a university center; Study 2: Unclear	English	Participants were given a packet of questionnaires and asked to complete and return them within a week.	8	?	Cholesterol-Saturated Fat Sub-scale	Nutrition Knowledge and Attitude Questionnaire, and the Connor Diet Habit Survey; The Connor Diet Habit Survey (DHS)
**Darawad et al (2017)**	Exercise Self-Efficacy scale (ESE-A)	Validation	272	52.9 ± 12.1	141M, 131F	Jordan	Cardiac, endocrine and renal outpatient clinics in 4 hospitals representing the private, public and university sectors	Arabic	Interview and participants could take the questionnaire home	18	?	No	?
**Everett, Salamonson, Davidson (2009)**	Bandura’s exercise self-efficacy scale	Validation	110	60.11 ± 10.57	79M, 31F	Australia	Cardiac Rehabilitation Services	English	Interview	18	?	No	6-Minute Walk Test, Borg Scale
**Fors et al (2014)**	Cardiac Self-Efficacy Scale (CSES Sweden)	Validation	288	62,2	216M, 72F	Sweden	Patients admitted to coronary units in hospitals in western Sweden	Swedish	Interview	12	?	Control the disease, Control symptoms and Maintain functionality	General self-efficacy; Fatalism
**Liu et al. (2021)**	Tai Chi Exercise Self-Efficacy scale (Mandarin version TCSE)	Validation	140	64.5 ± 8.1	53M, 87F	China	Community Health Center	Mandarin	Self-administered	14	?	TCSE Barriers and TCSE Performance	Total exercise time per week
**Mares et al (2020)**	Scale to Measure Self-efficacy and Self-management in People With Coronary Heart Disease (HH-SESM scale)	Development/ Validation	143	67.6 ± 11.8	91M, 52F	Australia	Private cardiology clinic, community centers, Cardiovascular Rehabilitation centers	English, Arabic	Interview	12	?	No	?
**Moseley(1999)**	Food Pyramid Self Efficacy Scale (FPSES)	Validation	30	70.4 ± 5.85	30M	USA	Clinic waiting room	English	Interview	22	?	No	Sickness Impact Profile
**Pedrosa and Rodrigues (2016); Pedrosa et al (2016)**	Self Efficacy for Appropriate Medication Adherence Scale (Brazilian version of the SEAMS)	Validation	147	59.9 ± 9.6	100M, 47F	Brazil	Cardiology outpatient clinic of a large university hospital in a city in the interior of São Paulo	BR Portuguese	Interview and consultation of medical records data	13	The sum of responses varies between 13 and 39; the higher the score, the greater the self-efficacy for adherence to medication treatment	Self-efficacy to take medication in difficult circumstances; Self-efficacy to continue taking medications when circumstances surrounding medication use are uncertain	Morisky Self-Reported Measure of Medication Adherence Scale (MMAS-4); General Perceived Self-Efficacy Scale (GSE)
**Rajati and Rajati (2019)**	Cardiac Exercise Self-efficacy Scale (Persian version CESE)	Validation	260	48.90 ± 13.77	141M, 119F	Iran	Imam Ali Cardiovascular Hospital	Persian	Interview	16	?	Knowledge, overcoming barriers, time management and recovery	The Cardiac Exercise Self-Efficacy scale; Exercise Self-Efficacy Scale; Hospital Anxiety and Depression Scale; Short Form-36 Health Survey Scale
**RISSER, JACOBSON, KRIPALANI et al (2007)**	Self-efficacy for Appropriate Medication Use (SEAMS)	Development/ Validation	436	63.8 ± 10.4	193M, 243F	USA	Emory University School of Medicine, Atlanta, GA	English	Interview	13	Potential scores for the 21-item scale ranged from 21 to 63. Higher scores indicated higher levels of self-efficacy for medication adherence	4 factors, but no nominations	Morisky’s selfreport measure of adherence; Rapid Estimate of Adult Literacy in Medicine; Wide Range Achievement Test-Revised
**Saengsiri, Thanaslip, Preechawong (2013)**	Cardiac Self-Efficacy Scale (CSES Thai version)	Validation	280	61.11 ± 10.94 with a range of 30–89 years.	206M, 74F	Thailand	Not reported	Thailand	Interview	14	?	Control symptoms, Maintain functionality	?
**Shajraw et al (2019)**	Cardiac Self-Efficacy Questionnaire (A-CSEQ)	Validation	268	57 years with a range of 38–82 years.	166M, 102F	Jordan	Cardiology ward	Arabic	The questionnaire was distributed and participants completed the CSEQ independently	16	?	Symptom control, Maintain functionality and Lifestyle	?
**Shin, Jang, Pender (2001)**	Exercise self-efficacy scale (for Korean)	Validation	249	18–29 years: 39.53 ± 16.29 30–59 years: 38.55 ± 18.48 >60 years:‡ 36.91 ± 25.08	?	South Korea	Hospitals and Health Centers in the South Korean cities of Seoul, Pusan, CheongJu, DaeJeon and Chuncheon and in the rural areas around CheongJu and Chunchon	South Korean	Interview	18	?	Interpersonal situation; Competing demands; Inner feelings	?
**Steca et al (2015)**	Cardiovascular Management Self-Efficacy Scale	Development/ Validation	172	66.4 ± 10.0 with variance of 38–86 years	131M, 41F	Italy	Three Italian Hospitals	Italian	Interview	9	?	Cardiac risk factor self-efficacy, therapy adherence self-efficacy, symptom recognition self-efficacy	Cardiovascular Management Self-Efficacy Scale; Sickness Impact Profile; Morisky Medication Adherence Scale; Scales of irritability and rumination; Cognitive Behavioral Assessment 2.0 Battery Italian; Brief-IPQ; NYHA
**Sullivan et al (1998)**	Cardiac Self-Efficacy Scale (CSES)	Development/ Validation	198	62.6 ± 8.9	164M, 34F	USA	Group Health Cooperative of Puget Sound and Group Health Northwest	English	Interview	13	?	Control symptoms, Maintain functionality	Hamilton Rating Scales for Depression; Hollingshead-Redlich social class score; Medical Outcomes Study SF-36; Sheehan’s Disability Scales; Jenkins Self-Efficacy Scales
**Taylor-Piliae and Froelicher (2004)**	Tai Chi exercise self-efficacy (TCSE Performance)	Development/ Validation	18	60.3 ± 18.4	5M, 13F	USA	Community center volunteers for seniors participating in Tai Chi exercise classes	Mandarin	Interview	14	A higher score indicates more confidence or perceived TCSE (0 = not at all confident, 100 = very confident)	TCSE Barriers and TCSE Performance	Total exercise time per week
**Wong et al. (2018)**	Self-Efficacy for Exercise Scale (Chinese version SEE-C)	Validation	355	51.1 ± 5.0	235M, 120F	China	Specialist clinics at two regional hospitals in Hong Kong	Mandarin	Participants completed the initial questionnaire, with assistance from a research assistant if requested.	9	?	No	Chinese version of the Hospital Anxiety and Depression Scale (HADS); Chinese (HK) version of the Health Survey Questionnaire Version 2 (SF-12v2; Total exercise time per week
**Zhang et al (2018)**	Cardiac Self-efficacy Scale (Chinese version C-CSES)	Validation	234	58.87 ± 10.97	169M, 55F	China	University-affiliated Hospital in Shiyan City, Hubei province, China	Mandarin	Interview	12	?	Control the disease, Control symptoms and Maintain functionality	Cardiac self-efficacy scale; General self-efficacy scale
**Zotti et al. (2007)**	General Perceived Self-Efficacy scale (GSE)	Validation	395	55.7 ± 9.6 with a range of 16–78 years	334M, 61F	Italy	Cardiac and Neurological Department of Hospital Rehabilitation Center	Italian	?	10	The total score for 10 items theoretically ranges from 10 to 40	No	?

USA: United States of America, (?): Not Reported

The included instruments assessed 8 different specific conditions that encompassed CAD in their participants, such as: 1) exercise self-efficacy: Cardiac Exercise Self-Efficacy Scale (Persian version CESE) [44], Exercise Self-Efficacy Scale (ESE) [37, 41, 43], Self-Efficacy for Exercise Scale Chinese version (SEE-C) [8]; 2) cardiac self-efficacy: Cardiac Self-Efficacy Scale (CSES) [12, 13, 35, 42, 45], and Scale to Measure Self-Efficacy and Self-Management in People With Coronary Heart Disease (HH-SESM Scale) [38]; 3) diet and nutrition self-efficacy: Cholesterol-Lowering Diet Self-Efficacy Scale [31], Food Pyramid Self Efficacy Scale (FPSES) [32]; 4) medication self-efficacy: Self-Efficacy for Appropriate Medication Use (SEAMS) [33, 46, 47]; 5) perceived general self-efficacy: General Perceived Self-Efficacy Scale (GSE) [40]; 6) cardiovascular management self-efficacy: Cardiovascular Management Self-Efficacy Scale [39]; 7) efficacy expectations: Barnason Efficacy Expectation Scale (BEES) [48]; 8) Tai Chi practice self-efficacy: Tai Chi Exercise Self-Efficacy (TCSE Performance) [34, 36].

Furthermore, the CSES [12, 13, 35, 42, 45] and ESE [37, 41, 43] showed the highest number of versions. Therefore, the CSES was considered the instrument with the broadest dissemination and is the oldest instrument included [12]. Regarding feasibility, the studies were not clear or did not provide data on factors such as time required for completion, instrument length, intellectual level required to respond, and facilitators. Concerning completion time, only the Arabic version of ESE [41] and the Thai CSES version [45] reported needing 20 and 15 minutes, respectively. No study reported whether a license is required for their use.

Although structural, content, and internal consistency validities were assessed in most studies, criterion, construct, cross-cultural validities, reliability, measurement error, and responsiveness were evaluated in a limited number of studies, either due to a complete or partial absence of data. Regarding reliability, the lack of data can negatively affect the reproducibility of consistent results. Measurement invariance was not assessed as no study presented such a property.

### Methodological quality of the studies

The data obtained from the methodological assessment of the studies are summarized in Table 2. Nine out of the ten clinimetric properties were evaluated, with the exception of measurement invariance, which was not mentioned by any study.

**Table 2:** Methodological assessment of measurement properties.

PROM (Reference)	Content validity	Structural validity	Internal consistency	Cross-cultural validity	Reliability	Measurement error	Criterion validity	Construct validity	Responsiveness
Barnason Efficacy Expectation Scale (BEES) [48]	D	A	V	N/A	I	I	V	D	I
Cardiac Exercise Self-Efficacy Scale Persian version (CESE) [44]	D	A	V	V	I	I	I	V	I
Cardiac Self-Efficacy Scale (A-CSEQ) [42]	A	V	V	V	I	I	I	A	I
Cardiac Self-efficacy Scale (C-CSES) [35]	V	V	V	V	I	I	I	D	I
Cardiac Self-Efficacy Scale (CSES Sweden) [13]	V	V	V	V	I	I	I	D	I
Cardiac Self-Efficacy Scale (CSES Thai version) [45]	V	V	V	V	I	I	I	D	I
Cardiac Self-Efficacy Scale (CSES) [12]	A	A	V	N/A	I	I	I	A	I
Cardiovascular Management Self-Efficacy Scale [39]	D	V	V	N/A	I	I	V	V	A
Cholesterol-Lowering Diet Self-Efficacy Scale [31]	I	I	V	N/A	I	I	V	I	I
Bandura’s Exercise Self-Efficacy Scale (Australian version) [37]	D	I	V	I	I	I	A	I	A
Bandura’s Exercise Self-Efficacy Scale (ESE-A) [41]	I	A	V	D	I	I	V	I	I
Bandura’s Exercise Self-Efficacy Scale (for Korean) [43]	D	A	V	V	I	I	I	A	I
Food Pyramid Self Efficacy Scale (FPSES) [32]	A	I	V	D	I	I	I	I	I
General Perceived Self-Efficacy Scale (GSE) [40]	I	I	V	I	I	I	I	I	I
Scale to Measure Self-Efficacy and Self-Management in People With Coronary Heart Disease (HH-SESM Scale) [38]	D	I	V	N/A	I	I	I	D	I
Self Efficacy for Appropriate Medication Adherence Scale (Brazilian version of the SEAMS) [46, 47]	D	I	V	V	I	I	V	I	I
Self-Efficacy for Appropriate Medication Use (SEAMS) [33]	D	A	V	N/A	I	I	V	D	I
Self-Efficacy for Exercise Scale Chinese Version (SEE-C) [8]	I	V	V	D	I	I	V	I	I
Tai Chi exercise Self-Efficacy Peformance (TCSE) [34]	I	I	V	V	I	D	I	I	I
Tai Chi Exercise Self-Efficacy Scale Mandarin version (TCSE) [36]	I	V	V	I	I	I	V	I	I

V = very good, A = adequate, D = doubtful, I = inadequate; N/A = not applicable.

The general requirements for development were satisfactorily met (e.g., clear description of the construct, clear description of the target population for which the instrument is intended, and its context of use). However, the studies either did not provide or did not demonstrate clarity in at least one requirement, leading to their classification as "inadequate" (Table 2).

Content validity was assessed as adequate in four studies: Chinese [35], Swedish [13], and Thai [45] versions of the CSES, and FPSES [32]. Eight studies showed unsatisfactory assessment or did not report the factorial evaluation of their instruments for structural validity [31, 32, 34, 37, 38, 40, 46, 47]. In the analysis of internal consistency, all studies were categorized as "very good," with Cronbach’s alpha values ranging from 0.68 to 0.97. Steca et al.’s study (2015) [39] was the only one that presented a value below α = 0.70 for one of the instrument domains [19]. Only nine studies reported following COSMIN recommendations for the cross-cultural adaptation phase of their instruments, and were considered "very good" [13, 34, 35, 42–47] (Table 2).

Regarding reliability, no study received a good methodological evaluation. All studies were considered "inadequate" in at least one of the eight assessed requirements. This is due to not reporting data on the Kappa agreement coefficient test and intraclass correlation coefficient.

Measurement error was deemed "adequate" only in the studies by Fors et al. (2014) [13] and Taylor-Piliae and Froelicher (2004) [34]. As for criterion validity, ten studies received a good assessment [8, 31, 33, 34, 36, 37, 39, 41, 45, 48].

The construct validity of the studies was conducted through hypothesis testing, mainly focusing on convergent and discriminant validity. Only one study reported parameters through divergent validity [44]. Nine studies were classified as "inadequate" due to lack of clarity or absence of measurement property data [8, 31, 32, 34, 36, 37, 40, 41, 46, 47]. For responsiveness, only two studies provided relevant information for this property and were considered "adequate" [37, 39].

### Summary of quality and level of evidence

Only one instrument demonstrated low quality in its internal consistency [39]. Content validity, structural validity, cross-cultural validity, criterion validity, and construct validity showed mixed qualities. Reliability, measurement error, and responsiveness exhibited low overall quality. Table 3 provides a summary of the evaluated measurement properties.

- *Bandura’s Exercise Self-Efficacy Scale (ESE)*: assessed in three versions adapted for distinct populations. Content validity was unsatisfactory in all versions. Structural validity was deemed unsatisfactory in the Australian version [37] (with factor loadings > 0.40). Internal consistency received an inadequate assessment in the South Korean version [43] for not providing Cronbach’s alpha for each domain. Cross-cultural validity and construct validity received mixed evaluations, and only the South Korean version [43] was considered three-dimensional. The overall quality of evidence for the instrument was mixed. Only internal consistency was deemed high. Structural, criterion, and construct validities were rated as moderate, and content validity was rated as low. Therefore, it was categorized as a level C recommendation.
- *Barnason Efficacy Expectation Scale (BEES)* [48]: Content validity was considered inconsistent. Structural validity (factor loading > 0.40), internal consistency (Cronbach’s alpha 0.92), and construct validity were deemed sufficient. The latter indicated that the instrument is unidimensional. The instrument provided insufficient data for reliability, measurement error, and responsiveness. The quality of evidence received mixed ratings, with internal consistency, structural validity, criterion validity, and construct validity rated as high. However, content validity was considered low. Therefore, it was categorized as a level B recommendation.
- *Cardiac Exercise Self-Efficacy Scale Persian Version (CESE)* [44]: Structural validity (factor loading > 0.45) and construct validity were considered sufficient. Its exploratory analysis identified a 4-factor structure (knowledge, overcoming barriers, time management, and recovery). However, it did not provide Cronbach’s alpha values for each domain, resulting in an inadequate rating. Cross-cultural validity was satisfactory. The quality of evidence for the instrument received mixed ratings, with structural validity, cross-cultural validity, and construct validity considered high, while internal consistency and content validity were rated low by the assessors. The instrument was categorized as a level C recommendation.
- *Cardiac Self-Efficacy Scale (CSES)*: The instrument was evaluated in its original version [12] and four adaptations for different populations. Content validity was considered satisfactory in all versions. Structural validity was considered inconsistent only in the original version [12] and satisfactory in all four adaptations [13, 42, 35, 45]. The original model [12] and the Arabic version [45] presented as bidimensional, while the other adapted versions presented as tridimensional models. The versions showed discrepancies in assessing internal consistency, as the Chinese [35] and Swedish [13] versions did not report individual Cronbach’s alpha values for their three domains. Construct validity received mixed evaluations. The quality of evidence for the instrument received mixed ratings, with high content validity, structural validity, cross-cultural validity, and internal consistency. The instrument was categorized as a level B recommendation.
- *Cardiovascular Management Self-Efficacy Scale* [39]: Structural validity (tridimensional model with factor loadings from 0.86 to 0.95), construct validity, criterion validity, and internal consistency received satisfactory ratings, but content validity obtained an insufficient rating. The quality of evidence for the instrument received mixed ratings, with only structural validity, criterion validity, construct validity, and internal consistency receiving high evaluations. The instrument was classified as a level B recommendation.
- *Cholesterol-Lowering Diet Self-Efficacy Scale* [31]: Content validity was considered inconsistent, structural validity (no data provided in the study) and construct validity were rated as unsatisfactory, while internal consistency (Cronbach’s alpha >0.93) and criterion validity were rated as satisfactory. The quality of evidence for the instrument received mixed ratings, with six properties considered low, including content validity and structural validity. Therefore, the instrument was classified as level C evidence.
- *Food Pyramid Self-Efficacy Scale (FPSES)* [32]: The instrument received satisfactory ratings for content validity and internal consistency (Cronbach’s alpha 0.92). However, structural validity (no factor analysis performed) and criterion validity were considered unsatisfactory. The quality of evidence for measurement properties received mostly low ratings, with high ratings only for content validity and internal consistency. Therefore, the instrument was classified as level C recommendation.
- *General Perceived Self-Efficacy Scale (GSE)* [40]: The validated instrument received unsatisfactory ratings for content, criterion, and construct validity. It received a satisfactory rating only for internal consistency (Cronbach’s alpha 0.85). Structural validity was considered inconsistent. The quality of evidence for measurement properties received mixed evaluations, but predominantly low, with high ratings only for cross-cultural validity and internal consistency. Therefore, the instrument was classified as level C recommendation.
- *Scale to Measure Self-Efficacy and Self-Management in People With Coronary Heart Disease (HH-SESM Scale)* [38]: The developed instrument received inconsistent ratings for content and structural validity, with a satisfactory rating only for internal consistency (Cronbach’s alpha 0.83 for self-efficacy subscale). In terms of evidence quality, the instrument received predominantly low ratings, including content and structural validity, with high rating only for internal consistency. Therefore, the instrument was classified as level C recommendation.
- *Self-Efficacy for Appropriate Medication Use (SEAMS)*: In the evaluation of the original version [33] of the instrument and the Brazilian version, found in two studies [46, 47], structural validity, criterion validity, and internal consistency were considered satisfactory. However, content validity for both versions was considered insufficient, but they differed in terms of construct validity assessment. The original version [33] was evaluated as unsatisfactory. The original study has factor loadings > 0.40 and is considered four-dimensional [33], while the Brazilian version is bidimensional [46, 47]. Regarding evidence quality, the grouped instrument received mixed evaluations, being rated as high only in terms of structural validity, cross-cultural validity, criterion validity, and internal consistency. Content validity was classified as low. Therefore, the instrument was classified as level B recommendation.
- *Self-Efficacy for Exercise Scale Chinese Version (SEE-C)* [8]: The instrument received an unsatisfactory rating for content validity, moderate ratings for cross-cultural and construct validity (factor loading > 0.64, and considered unidimensional). Structural validity, criterion validity, and internal consistency (Cronbach’s alpha > 0.90) received satisfactory evaluations. Regarding evidence quality, the instrument received mixed assessments, receiving high ratings only for structural validity, criterion validity, and internal consistency. Content validity was considered low. Therefore, the instrument was classified as a level C recommendation.
- *Tai Chi Exercise Self-Efficacy Performance (TCSE)*: The validated instruments received satisfactory ratings in terms of internal consistency (Cronbach’s alpha > 0.95) and construct validity (factor loading > 0.40, considered bidimensional). They received mixed ratings in terms of content validity (varying from insufficient for the US version [34] to unsatisfactory for the Chinese version [36]), structural validity, and cross-cultural validity. In terms of the quality assessment of aggregated evidence, the instrument received mixed ratings, with high ratings for construct validity and internal consistency. Content validity and structural validity were considered moderate. Therefore, the instrument was classified as a level C recommendation.

**Table 3:** Quality and level of clinimetric properties.

PROM	REFERENCE/ASSESSMENT	Content validity	Structural validity	Internal consistency	Cross-cultural validity	Reliability	Measurement error	Criterion validity	Construct validity	Responsiveness
*Barnason Efficacy Expectation Scale (BEES)*	Barnason et al (2002)	±	+	+	N/A	-	-	+	+	-
	**OVERALL RATING**	**±**	**+**	**+**	** N/A**	**-**	**-**	**+**	**+**	**-**
	**QUALITY OF EVIDENCE**	**Low**	**High**	**High**	N/A	**Low**	**Low**	**High**	**High**	**Low**
*Cardiac Exercise Self-efficacy Scale (Persian version CESE)*	Rajati, Rajati (2019)	±	+	-	+	-	-	-	+	-
	**OVERALL RATING**	**±**	**+**	**-**	**+**	**-**	**-**	**-**	**+**	**-**
	**QUALITY OF EVIDENCE**	**Low**	**High**	**Low**	**High**	**Low**	**Low**	**Low**	**High**	**Low**
*Cardiac Self-Efficacy Scale (CSES)*	Shajrawi et al (2019)	±	+	+	+	-	-	-	-	-
	Zhang et al (2018)	+	+	-	+	-	-	-	+	-
	Fors et al (2014)	+	+	-	+	-	-	-	±	-
	SAENGSIRI, THANASILP, PREECHAWONG (2013)	+	+	+	+	-	-	-	±	-
	SULLIVAN et al (1998)	±	±	+		-	-	-	+	-
	**OVERALL RATING**	**+**	**+**	**+**	**+**	**-**	**-**	**-**	**±**	**-**
	**QUALITY OF EVIDENCE**	**High**	**High**	**High**	**High**	**Low**	**Low**	**Low**	**Moderate**	**Low**
*Cardiovascular Management Self-efficacy Scale*	STECA et al (2015)	-	+	+	N/A	-	-	+	+	+
	**OVERALL RATING**	**-**	**+**	**+**	** N/A**	**-**	**-**	**+**	**+**	**+**
	**QUALITY OF EVIDENCE**	**Low**	**High**	**High**	** N/A**	**Low**	**Low**	**High**	**High**	**Moderate**
*Cholesterol-Lowering Diet Self-Efficacy Scale*	Burke et al. (2003)	±	-	+	N/A	-	-	+	-	-
	**OVERALL RATING**	**±**	**-**	**+**	** N/A**	**-**	**-**	**+**	**-**	**-**
	**QUALITY OF EVIDENCE**	**Low**	**Low**	**High**	** N/A**	**Low**	**Low**	**High**	**Low**	**Low**
*Exercise self-efficacy scale (ESE)*	Everett, Salamonson, Davidson (2009)	±	-	+	-	-	-	+	-	+
	DARAWAD et al (2017)	±	+	+	±	-	-	+	±	-
	SHIN, JANG, PENDER (2001)	±	+	-	+	-	-	-	+	-
	**OVERALL RATING**	**±**	**±**	**+**	**±**	**-**	**-**	**±**	**±**	**-**
	**QUALITY OF EVIDENCE**	**Low**	**Moderate**	**High**	**Moderate**	**Low**	**Low**	**Moderate**	**Moderate**	**Low**
*Food Pyramid Self Efficacy Scale (FPSES)*	MOSELEY (1999)	+	-	+	±	-	-	-	±	-
	**OVERALL RATING**	**+**	**-**	**+**	**±**	**-**	**-**	**-**	**±**	**-**
	**QUALITY OF EVIDENCE**	**High**	**Low**	**High**	**Moderate**	**Low**	**Low**	**Low**	**Moderate**	**Low**
*General Perceived Self-Efficacy scale (GSE)*	Zotti et al. (2007)	-	±	+	-	-	-	-	-	-
	**OVERALL RATING**	**-**	**±**	**+**	**-**	**-**	**-**	**-**	**-**	**-**
	**QUALITY OF EVIDENCE**	**Low**	**Moderate**	**High**	**High**	**Low**	**Low**	**Low**	**Low**	**Low**
*Scale to Measure Self-efficacy and Self-management in People With Coronary Heart Disease (HH-SESM scale)*	Mares et al (2020)	±	-	+	N/A	-	-	-	±	-
	**OVERALL RATING**	**±**	**-**	**+**	**N/A**	**-**	**-**	**-**	**±**	**-**
	**QUALITY OF EVIDENCE**	**Low**	**Low**	**High**	**N/A**	**Low**	**Low**	**Low**	**Moderate**	**Low**
*Self-efficacy for Appropriate Medication Use (SEAMS)*	Pedrosa and Rodrigues (2016)	±	+	+	+	-	-	+	+	±
	Pedrosa et al (2016)	±	+	+	+	-	-	+	+	±
	RISSER, JACOBSON, KRIPALANI et al (2007)	±	+	+		-	-	+	-	±
	**OVERALL RATING**	**±**	**+**	**+**	**+**	**-**	**-**	**+**	**±**	**±**
	**QUALITY OF EVIDENCE**	**Low**	**High**	**High**	**High**	**Low**	**Low**	**High**	**Moderate**	**Low**
*Self-Efficacy for Exercise Scale (Chinese version SEE-C)*	Wong et al. (2018)	-	+	+	±	-	-	+	-	-
	**OVERALL RATING**	**-**	**+**	**+**	**±**	**-**	**-**	**+**	**-**	**-**
	**QUALITY OF EVIDENCE**	**Low**	**High**	**High**	**Moderate**	**Low**	**Low**	**High**	**Low**	**Low**
*Tai Chi exercise self-efficacy (TCSE Performance)*	Taylor-Piliae and Froelicher (2004)	±	-	+	+	-	-	-	±	±
	Liu et al. (2021)	-	+	+	-	-	-	+	+	±
	**OVERALL RATING**	**±**	**±**	**+**	**±**	**-**	**-**	**±**	**+**	**±**
	**QUALITY OF EVIDENCE**	**Moderate**	**Moderate**	**High**	**Moderate**	**Low**	**Low**	**Moderate**	**High**	**Moderate**

Adequate (+), inadequate (-), inconsistent (±), Not Applied (N/A).

### Linking of items from measurement instruments with the international classification of functioning, disability and health

Table 4 presents the results of linking the extracted items from the measurement instruments to the ICF. It was not possible to link the FPSES instrument [32], as it is not available in its study or on the web. We did not receive a response from the authors after email contact. Only five items could not be linked as they corresponded to personal factors and were present in the versions of the ESE instrument [37, 41, 43], and the HH-SESM Scale [38].

**Table 4:** Linkage of content extracted from instrument items to ICF codes.

INSTRUMENTS	AUTHOR (YEAR)	SUBDIMENSIONS	LINKED CATEGORIES	CODES	N
*Barnason Efficacy Expectation Scale (BEES)*	Barnason et al (2002)	No	(b) Body Functions, (s) Body Structures, (d) Activity and Participation, (e) Environmental Factors	b16710, b455, b1266, b28011, b280, b11420, s4100, d230, d640, d2302, d920, d9204, d760, d770, d2408, d5701, d599, d540, e355, e320, e1100, e1101, e110	23
*Cardiac Exercise Self-Efficacy Scale Persian Version (CESE)*	Rajati, Rajati (2019)	Knowledge, overcoming barriers, time management, and recovery	(b) Body Functions	b1148, b280, b28011, b410, b4100, b455	6
*Cardiac Self-Efficacy Scale (CSES)*	Shajraw et al (2019)	Symptom control, maintaining functionality, and lifestyle	(b) Body Functions, (s) Body Structures, (d) Activity and Participation, (e) Environmental Factors	b1670, b280, b28011, b410, b4100, b440, b4401, b455, b4551, b460, b530, b640, s4100, d2303, d570, d5701, d7609, d7702, d859, d920, e110, e1101, e310, e315, e355	25
	Zhang et al (2018)	Disease management, symptom control, and maintaining functionality	(b) Body Functions, (s) Body Structures, (d) Activity and Participation, (e) Environmental Factors	b1670, b280, b28011, b410, b4100, b440, b4401, b455, b4551, b460, s4100, d2303, d570, d7609, d859, d920, e110, e1101, e310, e315, e355	21
	Fors et al (2014)	Disease management, symptom control, and maintaining functionality	(b) Body Functions, (s) Body Structures, (d) Activity and Participation, (e) Environmental Factors	b1670, b280, b28011, b410, b4100, b440, b4401, b455, b4551, b460, b640, s4100, d2303, d570, d7609, d7702, d859, d920, e110, e1101, e310, e315, e355	23
	Saengsiri, Thanasilp, Preechawong (2013)	Symptom control, maintaining functionality	(b) Body Functions, (s) Body Structures, (d) Activity and Participation, (e) Environmental Factors	b1670, b280, b28011, b410, b4100, b440, b4401, b455, b460, b640, s4100, d2303, d240, d570, d7609, d7702, d859, d920, e110, e1101, e310, e315, e355	23
	Sullivan et al (1998)	Symptom control, maintaining functionality	(b) Body Functions, (s) Body Structures, (d) Activity and Participation, (e) Environmental Factors	b1670, b280, b28011, b410, b4100, b440, b4401, b455, b4551, b460, b640, s4100, d2303, d570, d7609, d7702, d859, d920, e110, e1101, e310, e315, e355	23
*Cardiovascular Management Self-Efficacy Scale*	Steca et al (2015)	Self-efficacy for cardiac risk factors, self-efficacy for therapy adherence, self-efficacy for symptom recognition	(b) Body Functions, (d) Activity and Participation, (e) Environmental Factors	b144, b1646, b280, b28011, b4100, b4101, b455, b4558, d2303, d240, d2401, d5701, d599, e110, e1100, e1101, e355	17
*Cholesterol-Lowering Diet Self-Efficacy Scale*	Burke et al. (2003)	Cholesterol-Saturated Fat Sub-scale	(b) Body Functions, (d) Activity and Participation, (e) Environmental Factors	b1266, b152, d5701, d240, d760, d7501, d855, d856, e1100, e325	10
*Bandura’s Exercise Self-Efficacy Scale (ESE)*	Darawad et al (2017)	No	(b) Body Functions, (d) Activity and Participation, (e) Environmental Factors	b152, b1642, b1646, b455, b4552, d760, d779, d850, d859, d920, e225, e245, e310, e315, e320, e325	16
	Everett, Salamonson, Davidson (2009)	No	(b) Body Functions, (d) Activity and Participation, (e) Environmental Factors	b152, b1642, b1646, b455, b4552, d760, d779, d850, d859, d920, e225, e245, e310, e315, e320, e325	16
	Shin, Jang, Pender (2001)	Interpersonal situation; Concurrent demands; Internal feelings	(b) Body Functions, (d) Activity and Participation, (e) Environmental Factors	b152, b1642, b1646, b455, b4552, d760, d779, d850, d859, d920, e225, e245, e310, e315, e320, e325	16
*General Perceived Self-Efficacy scale (GSE)*	Zotti et al. (2007)	No	(b) Body Functions, (d) Activity and Participation	b175, b1646, b1265, b1266, d240	5
*Scale to Measure Self-Efficacy and Self-Management in People With Coronary Heart Disease (HH-SESM Scale)*	Mares et al (2020)	No	(b) Body Functions, (d) Activity and Participation, (e) Environmental Factors	b1642, b455, b530, d2303, d240, d5701, e1100, e1101, e355	9
*Self-Efficacy for Appropriate Medication Use (SEAMS)*	Pedrosa e Rodrigues (2016); Pedrosa et al (20216)	Self-efficacy to take medications under difficult circumstances; Self-efficacy to continue taking medications when circumstances involving medication use are uncertain	(b) Body Functions, (d) Activity and Participation, (e) Environmental Factors	b1641, b144, b1642, b1140, b460, d9208, d2200, d2301, e1101, e110, e1108, e355	12
	Risser, Jacobson, ***Kripalani*** (2007)	4 factors, but without designations	(b) Body Functions, (d) Activity and Participation, (e) Environmental Factors	b1266, b144, b460, b1641, b1140, b1642, d9208, d2200, d2301, e110, e1101, e355, e1108	13
*Self-Efficacy for Exercise Scale Chinese version (SEE-C)*	Wong et al. (2018)	No	(b) Body Functions, (d) Activity and Participation, (e) Environmental Factors	b1301, b152, b1642, b2408, b280, b28011, b455, b4552, d2301, d2401, e225, e245	12
*Tai Chi Exercise Self-Efficacy Peformance (TCSE)*	Liu et al. (2021)	TCSE Barriers e TCSE Performance	(b) Body Functions, (d) Activity and Participation, (e) Environmental Factors	b455, b1301, b152, b280, b28011, b1641, b1642, d2401, d240, d4558, d4559, e245, e225	13
	Taylor-Piliae and Froelicher (2004)	TCSE Barriers e TCSE Performance	(b) Body Functions, (d) Activity and Participation, (e) Environmental Factors	b455, b1301, b152, b280, b28011, b1641, b1642, d2401, d240, d4558, d4559, e245, e225	13

A total of 321 concepts were identified from the 276 items of the 19 instruments. Regarding the process of linking to the ICF, categories were related to the majority of the instruments (originals and their adapted versions). The component body functions (b) was linked to all 19 instruments, the component body structures (s) was linked to 6 instruments, the component activities and ***participation*** (d) was linked to 18 instruments, and finally, the component environmental factors (e) was linked to 17 instruments. The chapters with the highest number of links were the chapters on mental functions (b1) and functions of the cardiovascular, hematological and immunological systems, and the respiratory system (b4).

The instrument with the highest number of concept links to the ICF was the Cardiac Self-Efficacy Scale (CSES), although there was a slight divergence in its 5 versions included in the study, with the Arabic version [33] having 25 (the highest number of linkages), the original [12], Swedish [41], and Thai [44] versions having 23, and the Chinese version having the lowest number of linkages, totaling 21.

## Discussion

This systematic review identified twenty-one studies regarding the development and/or validation of 12 instruments that assess cardiac self-efficacy, medication, exercise, cardiac surgery, rehabilitation, nutrition, lifestyle, and risk factors in subjects with CAD. Instruments were classified as evidence levels B and C. Among the included instruments the CSES was the most disseminated as well as the instrument with the highest number of concept links to the ICF.

Following the COSMIN guidelines, all studies exhibit methodological failures in several significant information regarding measurement properties. These findings corroborate with a systematic review performed by Frei et al (2009) [49], in which they identified a large number of self-efficacy instruments for subjects with chronic diseases. However, all of them demonstrated significant limitations in their development and validation [49].

Considering the modified GRADE [19] no instrument was classified as evidence level A. The BEES, CSES, SEAMS, and CMSES were categorized as level B, suggesting they may be recommended for use when assessing the target population. A systematic review performed by Kavradim et al (2020) [50] focusing solely on self-efficacy instruments for cardiac purposes in general population with cardiovascular diseases found similar results which corroborates with our findings to the CSES and CMSES. Another review performed by Lamarche, Tejpal, and Mangin (2018) [51] assessing self-efficacy instruments in medication management found that SEAMS instrument [33] is the most appropriate self-efficacy scale.

The validity of content, considered the most important measurement property of an instrument according to COSMIN [26], is evaluated in relation to its target population, data collection, moderator, interviews, recording, transcription, sample size, and data analysis [26]. It was observed that the majority of studies presented inconsistent data on the assessed items [8, 31, 33, 34, 36, 38–44, 46–48]. These results are similar with the review by Kavradim et al (2020), which classified CSES with high quality and CMSES with low quality for validity content [50].

Structural validity provides evidence through exploratory factor analysis (EFA) and confirmatory factor analysis (CFA) [19]. However, such data were not found in some instruments [31, 32, 34, 38], leading to their downgraded classifications. The CSES and CMSES instruments demonstrated high quality for structural validity, consistent with the review by Kavradim et al (2020) [50]. It was also observed divergence in dimensions and item numbers between the different versions of CSES [12, 13, 35, 42, 45] and ESE [37, 41, 43]. These differences may be related to cultural, educational, and socioeconomic factors of each country.

All studies measured internal consistency using Cronbach’s alpha, but only a minority conducted test-retest reliability analyses. These data corroborate with the findings of Frei et al (2009) [49], Kavradim et al (2020) [50], and Lamarche, Tejpal, and Mangin (2018) [51]. We recommend that validation processes include relevant tests to the instrument’s purpose, and every validation should incorporate test-retest reliability analysis, preferably using intraclass correlation coefficients [25].

Regarding the ICF, the CSES instrument in all its versions showed the highest number of linkages of its items with the four ICF categories and codes [50]. This may be related to the diversity of content assessed in its items, which can increase the range of codes, making it the most comprehensive instrument included in the review. As the ICF classification is a reference in clinical practice, teaching, and research language, this data becomes relevant [29, 50].

All item contents that could not be linked to the ICF referred to factors not yet covered by it and were present in all ESE instrument versions [37, 41, 43], and the HH-SESM Scale [38]. This reinforces the reported importance of identifying these contents in linkage studies to strengthen the inclusion of additional factors in the ICF in the future [51, 52].

The findings of this review show that all instruments assessing self-efficacy for individuals with CAD have some shortcomings in their measurement properties. Therefore, it is recommended to develop more robust self-efficacy instruments for individuals with CAD with fewer biases that could compromise the measured results.We believe that the results of this review can contribute to the selection of appropriate instruments for assessing self-efficacy levels in CAD in different contexts.

Moreover, considering the evidence level found in this review, we consider that the use of such instruments in clinical practice, research and teaching may be carefully assessed as improvements in measurement properties are needed for better evaluation. In this way, the choice of health professionals, researchers and academics should be based on validated instruments according to the target population as well as those with appropriate measurement properties and greater content linkage with the ICF for language standardization.

It is worth highlighting that this is the first systematic review assessing self-efficacy instruments for CAD as well as linking self-efficacy instruments to the ICF. This study was also conducted in accordance with the recommendations of Cochrane [18] and COSMIN [19, 25] by two independent authors without any language or time restriction. Furthermore, the PRISMA 2020 Main Checklist was adopted resulting in a more transparent, comprehensive, and accurate review (S1 Checklist). Although we have limited the inclusion criteria to self-efficacy instruments for coronary patients, the heterogeneity of the included instruments may have made the discussion of measurement properties more challenging. Another limitation may be related to the lack of patient and public involvement in its development.

## Conclusion

Despite the large number of instruments assessing self-efficacy in individuals with CAD, none of them showed strong properties regarding the procedures adopted for their development and measurement validity. The best evidence level found was categorized as B which means a potential to be recommended. However, further clinimetric studies in accordance with COSMIN are required for evaluating self-efficacy in individuals with CAD. Regarding the linkage with the ICF, CSES had the highest number of linkages with ICF codes in the categories of body functions and structures, activities and participation, and environmental factors. In this way, the CSES may be considered the most comprehensive instrument assessed in this study, considering the importance of the ICF in standardizing clinical language.

## Supporting information

S1 Checklist PRISMA checklist. (DOCX)

S1 File Search strategy. (DOCX)

S1 Data (XLSX)
