# Supplementary material for: Self-efficacy measurement instruments for individuals with coronary artery disease: A systematic review
Source: PLoS One. 2024 Mar 4;19(3):e0299041. doi: 10.1371/journal.pone.0299041 (PMC10911622; doi:10.1371/journal.pone.0299041)
Supplement: S1 File — (DOCX) [file pone.0299041.s002.docx]

**SUPPLEMENTARY FILE 1**

**Search strategy for MEDLINE (Ovid)**

1. exp Self Efficacy/

2. self-efficacy.mp.

3. 1 or 2

4. exp Coronary Artery Disease/

5. Coronary Artery Disease.mp.

6. coronary heart disease.mp.

7. exp Coronary Disease/

8. exp Acute Coronary Syndrome/

9. Heart disease.mp. [mp=title, abstract, original title, name of substance word, subject heading word, floating sub-heading word, keyword heading word, organism supplementary concept word, protocol supplementary concept word, rare disease supplementary concept word, unique identifier, synonyms]

10. exp heart diseases/

11. exp myocardial ischemia/

12. exp Cardiovascular Diseases/

13. Cardiovascular Disease.mp.

14. exp Heart Failure/

15. heart failure.mp.

16. 4 or 5 or 6 or 7 or 8 or 9 or 10 or 11 or 12 or 13 or 14 or 15

17. instrument*.mp.

18. instruments*.mp.

19. measure*.mp.

20. measures*.mp.

21. questionnaire*.mp.

22. exp "Surveys and Questionnaires"/

23. questionnaires*.mp.

24. scale*.mp. [mp=title, abstract, original title, name of substance word, subject heading word, floating sub-heading word, keyword heading word, organism supplementary concept word, protocol supplementary concept word, rare disease supplementary concept word, unique identifier, synonyms]

25. scales*.mp. [mp=title, abstract, original title, name of substance word, subject heading word, floating sub-heading word, keyword heading word, organism supplementary concept word, protocol supplementary concept word, rare disease supplementary concept word, unique identifier, synonyms]

26. tool*.mp. [mp=title, abstract, original title, name of substance word, subject heading word, floating sub-heading word, keyword heading word, organism supplementary concept word, protocol supplementary concept word, rare disease supplementary concept word, unique identifier, synonyms]

27. tools*.mp.

28. survey*.mp.

29. test*.mp.

30. 17 or 18 or 19 or 20 or 21 or 22 or 23 or 24 or 25 or 26 or 27 or 28 or 29

31. 3 and 16 and 30

32. (instrumentation or methods).fs.

33. (Validation Studies or Comparative Study).pt.

34. exp Psychometrics/

35. psychometr*.ti,ab.

36. (clinimetr* or clinometr*).tw.

37. outcome assessment.ti,ab.

38. outcome measure*.tw.

39. exp Observer Variation/

40. observer variation.ti,ab.

41. exp Health Status Indicators/

42. exp Reproducibility of Results/

43. reproducib*.ti,ab.

44. exp Discriminant Analysis/

45. (reliab* or unreliab* or valid* or coefficient or homogeneity or homogeneous or internal consistency).ti,ab.

46. (cronbach* and (alpha or alphas)).ti,ab.

47. (item and (correlation* or selection* or reduction*)).ti,ab.

48. (agreement or precision or imprecision or precise values or test-retest).ti,ab.

49. (test and retest).ti,ab.

50. (reliab* and (test or retest)).ti,ab.

51. ((replicab* or repeated) and (measure or measures or findings or result or results or test or tests)).ti,ab.

52. (generaliza* or generalisa* or concordance).ti,ab.

53. (intraclass and correlation*).ti,ab.

54. (discriminative or known group or factor analysis or factor analyses or dimension* or subscale*).ti,ab.

55. (multitrait and scaling and (analysis or analyses)).ti,ab.

56. (item discriminant or interscale correlation* or error or errors or individual variability).ti,ab.

57. (variability and (analysis or values)).ti,ab.

58. (uncertainty and (measurement or measuring)).ti,ab.

59. (standard error of measurement or sensitiv* or responsive*).ti,ab.

60. ((minimal or minimally or clinical or clinically) and (important or significant or detectable) and (change or difference)).ti,ab.

61. (small* and (real or detectable) and (change or difference)).ti,ab.

62. (meaningful change or ceiling effect or floor effect or Item response model or IRT or Rasch or Differential item functioning or DIF or computer adaptive testing or item bank or cross-cultural equivalence).ti,ab.

63. exp Reproducibility of Results/

64. cross-cultural equivalence.ti,ab.

65. development.ti,ab.

66. 32 or 33 or 34 or 35 or 36 or 37 or 38 or 39 or 40 or 41 or 42 or 43 or 44 or 45 or 46 or 47 or 48 or 49 or 50 or 51 or 52 or 53 or 54 or 55 or 56 or 57 or 58 or 59 or 60 or 61 or 62 or 63 or 64 or 65

67. 31 and 66

**Search strategy for Web of Science**

| *n* | Search terms |
| --- | --- |
| #1 | Search: TS=(Self-Efficacy*) OR TS=(Self Efficacy*) |
| #2 | Search: TS=(Coronary Artery Disease*)  OR TS=(Coronary Disease*) OR TS=(Coronary Heart Disease*) OR TS=(Acute Coronary Syndrome) OR TS=(Heart disease*) OR TS=(Cardiovascular disease*) OR TS=(Heart failure) OR TS=(Myocardial ischemia) |
| #3 | Search: TS=(Instrument*) OR TS=(instruments*) OR TS=(measure*) OR TS=(measures*) OR TS=(questionnaire*) OR TS=(questionnaires*) OR TS=(scale*) OR TS=(scales*) OR TS=(tool*) OR TS=(tools*) OR TS=(survey*) OR TS=(test*) |
| #4 | Search: TS=(instrumentation) OR TS=(methods) OR TS=(“validation stud*”) OR TS=(“comparative stud*”) OR TS=(psychometrics) OR TS=(psychometr*) OR ALL=(clinimetr*) OR ALL=(clinometr*) OR TS=(“outcome assessment”) OR TS=(“outcome measure”) OR TS=(“observer variation”) OR TS=(“observer variation”) OR TS=(“health status indicators”) OR TS=(“reproducib*”) OR TS=(“discriminant analysis”) OR TS=(reliab*) OR TS=(unreliab*) OR TS=(valid*) OR TS=(“coefficient of variation”) OR TS=(coefficient) OR TS=(homogeneity) OR TS=(homogeneous) OR TS=(“internal consistency”) OR ((TS=(alpha) OR TS=(alphas)) AND TS=(cronbach*)) OR ((TS=(correlation*) OR TS=(selection*) OR TS=(reduction*)) AND TS=(item)) OR TS=(agreement) OR TS=(precision) OR TS=(imprecision) OR TS=(precise values) OR TS=(test-retest) OR (TS=(test) AND TS=(retest)) OR ((TS=(test) OR TS=(retest)) AND TS=(reliab*)) OR TS=(stability) OR TS=(interrater) OR TS=(inter-rater) OR TS=(intrarater) OR TS=(intra-rater) OR TS=(intertester) OR TS=(inter-tester) OR TS=(intratester) OR TS=(intra-tester) OR TS=(interobserver) OR TS=(inter-observer) OR TS=(intraobserver) OR TS=(intra-observer) OR TS=(intertechnician) OR TS=(inter-technician) OR TS=(intratechnician) OR TS=(intra-technician) OR TS=(interexaminer) OR TS=(inter-examiner) OR TS=(intraexaminer) OR TS=(intra-examiner) OR TS=(interassay) OR TS=(inter-assay) OR TS=(intraassay) OR TS=(intra-assay) OR TS=(interindividual) OR TS=(inter-individual) OR TS=(intraindividual) OR TS=(intra-individual) OR TS=(interparticipant) OR TS=(inter-participant) OR TS=(intraparticipant) OR TS=(intra-participant) OR TS=(kappa) OR TS=(kappa’s) OR TS=(kappas) OR TS=(repeatab*) OR ((ALL=(replicab*) OR ALL=(repeated)) AND (ALL=(measure) OR ALL=(measures) OR ALL=(findings) OR ALL=(result) OR ALL=(results) OR ALL=(test) OR ALL=(tests))) OR TS=(generaliza*) OR TS=(generalisa*) OR TS=(concordance) OR (TS=(intraclass) AND TS=(correlation*)) OR TS=(discriminative) OR TS=(known group) OR TS=(“factor analysis”) OR TS=(“factor analyses”) OR TS=(“factor structure”) OR TS=(“factor structures”) OR TS=(dimension*) OR TS=(subscale*) OR ((TS=(analysis) OR TS=(analyses)) AND TS=(scaling) AND TS=(multitrait)) OR TS=(“item discriminant”) OR TS=(“interscale correlation*”) OR TS=(error) OR TS=(errors) OR TS=(“individual variability”) OR TS=(“interval variability”) OR TS=(“rate variability”) OR ((TS=(values) OR TS=(analysis)) AND TS=(variability)) OR ((TS=(measurement) OR TS=(measuring)) AND TS=(uncertainty)) OR TS=(“standard error of measurement”) OR TS=(sensitiv*) OR TS=(responsive*) OR (TS=(limit) AND TS=(detection)) OR TS=(“minimal detectable concentration”) OR TS=(interpretab*) OR ((TS=(minimal) OR TS=(minimally) OR TS=(clinical) OR TS=(clinically)) AND (TS=(important) OR TS=(significant) OR TS=(detectable)) AND (TS=(change) OR TS=(difference))) OR (TS=(small) AND (TS=(real) OR TS=(detectable)) AND (TS=(change) OR TS=(difference))) OR TS=(“meaningful change”) OR TS=(“ceiling effect”) OR TS=(“floor effect”) OR TS=(“Item response model”) OR TS=(IRT) OR TS=(Rasch) OR TS=(“differential item functioning”) OR TS=(DIF) OR TS=(“computer adaptive testing”) OR TS=(“item bank”) OR TS=(“cross-cultural equivalence”) OR TS=(“development”) |
| #5 | #1 AND #2 AND #3 AND #4 |

**Search strategy for Embase and PsycoINFO**

| *n* | Search terms |
| --- | --- |
| #1 | (“Self-Efficacy*” OR “Self Efficacy*”) |
| #2 | (“Coronary Artery Disease*”  OR “Coronary Disease*” OR “Coronary Heart Disease*” OR “Acute Coronary Syndrome” OR “Heart disease*” OR “Cardiovascular disease*” OR “Heart failure” OR “Myocardial ischemia”) |
| #3 | (“Instrument*” OR “Instruments*” OR “measure*” OR “measures*” OR “questionnaire*” OR “questionnaires” OR “scale*” OR “scales*” OR “tool*” OR “tools*” OR  “survey*” OR “test*”) |
| #4 | ("instrumentation” OR “methods” OR “Validation Studies” OR “Comparative Study” OR “psychometrics” OR “psychometr*” OR “clinimetr*” OR “clinometr*” OR “outcome assessment (health care)” OR “outcome assessment” OR “outcome measure*” OR “observer variation” OR “observer variation” OR “Health Status Indicators” OR “reproducibility of results” OR “reproducib*” OR “discriminant analysis” OR “reliab*” OR “unreliab*” OR “valid*” OR “coefficient of variation” OR “coefficient” OR “homogeneity” OR “homogeneous” OR “internal consistency” OR (“cronbach*” AND “alpha” OR “alphas”) OR (“item” AND (“correlation*” OR “selection*” OR “reduction*”)) OR “agreement” OR “precision” OR “imprecision” OR “precise values” OR “test-retest” OR (“test” AND “retest”) OR (“reliab*” AND (“test” OR “retest”)) OR “stability” OR “interrater” OR “inter-rater” OR “intrarater” OR “intra-rater” OR “intertester” OR “inter-tester” OR “intratester” OR “intra-tester” OR “interobserver” OR “inter-observer” OR “intraobserver” OR “intra-observer” OR “intertechnician” OR “inter-technician” OR “intratechnician” OR “intra-technician” OR “interexaminer” OR “inter-examiner” OR “intraexaminer” OR “intra-examiner” OR “interassay” OR “inter-assay” OR “intraassay” OR “intra-assay” OR “interindividual” OR “inter-individual” OR “intraindividual” OR “intra-individual” OR “interparticipant” OR “inter-participant” OR “intraparticipant” OR “intra-participant” OR “kappa” OR “kappa’s” OR “kappas” OR “repeatab*” OR ((“replicab*” OR “repeated”) AND (“measure” OR “measures” OR “findings” OR “result” OR “results” OR “test” OR “tests”)) OR “generaliza*” OR “generalisa*” OR “concordance” OR (“intraclass” AND “correlation*”) OR “discriminative” OR “known group” OR “factor analysis” OR “factor analyses” OR “factor structure” OR “factor structures” OR “dimension*” OR “subscale*” OR (“multitrait” AND “scaling” AND (“analysis” OR “analyses”)) OR “item discriminant” OR “interscale correlation*” OR “error” OR “errors” OR “individual variability” OR “interval variability” OR “rate variability” OR (“variability” AND (“analysis” OR “values”)) OR (“uncertainty” AND (“measurement” OR “measuring”)) OR “standard error of measurement” OR “sensitiv*” OR “responsive*” OR (“limit” AND “detection”) OR “minimal detectable concentration” OR “interpretab*” OR ((“minimal” OR “minimally” OR “clinical” OR “clinically”) AND (“important” OR “significant” OR “detectable”) AND (“change” OR “difference”)) OR (“small*” AND (“real” OR “detectable”) AND (“change” OR “difference”)) OR “meaningful change” OR “ceiling effect” OR “floor effect” OR “Item response model” OR “IRT” OR “Rasch” OR “Differential item functioning” OR “DIF” OR “computer adaptive testing” OR “item bank” OR “cross-cultural equivalence” OR “development”) |
| #5 | #1 AND #2 AND #3 AND #4 |
